# Coriander (*Coriandrum sativum*) Polyphenols and Their Nutraceutical Value against Obesity and Metabolic Syndrome

**DOI:** 10.3390/molecules28104187

**Published:** 2023-05-19

**Authors:** Samir Scandar, Claudia Zadra, Maria Carla Marcotullio

**Affiliations:** Department of Pharmaceutical Sciences, University of Perugia, Via del Giochetto-Ed. B, 06122 Perugia, Italy; samir.scandar@studenti.unipg.it (S.S.); claudia.zadra@unipg.it (C.Z.)

**Keywords:** *Coriandrum sativum* L., polyphenols, antioxidant activity, anti-inflammatory activity, obesity, metabolic syndrome, diabetes

## Abstract

Coriander is a widely used plant for its medicinal and biological properties. Both coriander essential oil and extracts are interesting sources of bioactive compounds and are widely used as spices in culinary practice due to their exclusive aroma and flavour. We focus our attention on coriander extracts that are rich in polyphenols. It is well known that plant polyphenols possess different biological activities and several functional foods contain this class of compounds. The polyphenol profile in an extract can be influenced by the plant part studied, the method of extraction and other parameters. This study performs a literature review using the words “coriander”, “polyphenols” and “extraction” or “biological activity” in different databases such as PubMed, Google Scholar and Scopus. After that, we focus on the evidence of coriander polyphenols as protective agents against some inflammation-related diseases. Due to the bioactivities of coriander extract, this herb can be considered a valuable functional food against obesity, metabolic syndrome and diabetes.

## 1. Introduction

Coriander (*Coriandrum sativum* L., Apiaceae) was first cited in the Ebers papyrus (1550 BC) [1] and is used in cuisine and traditional medicines. It is mainly cultivated for its yellowish-brown globular fruits, known as coriander seeds [2,3,4]. Originating from the Mediterranean and Middle Eastern regions, this fast-growing annual herbaceous and domestic plant is also found in South America, North Africa and India [5,6,7].

The genus comprises only two known species: *C. sativum* L. and *C. tordylium* (Fenzl) Bornm, or wild coriander [1], although the WFO Plant List [8] reports two other species (*C. digitatum* DC and *C. maritimum* L.) which are still not checked and waiting for taxonomic scrutiny. The plant is characterised by its monochasial and branched stem, reaching a maximum height of 50 cm, with pinkish or whitish-coloured small flowers arranged in an umbel-like inflorescence. The etymology of the term “coriandrum” derived from the Greek words “*koriannon*”, meaning stink bug, and “*annon*” which means “fragrant anise”, as the plant emits a distinct odour as it matures [1,9,10].

Coriander leaves are known as cilantro and Chinese parsley, and are used in many countries as a common flavouring agent [9], a condiment and a food preservative in the food industry [11]. Coriander seeds are the common spice “coriander” and are used to prepare beverages, in particular in gin production [12], and sometimes it is used to mask odd flavours [13].

Coriander is recognised for its medicinal properties [14] and in traditional medicine, it has been used as a carminative to treat dyspepsia, flatulence and diarrhoea, to relieve respiratory and urinary problems, but also as an antiemetic [15]. The biological activities of coriander have been largely studied due to their potential health benefits. Coriander’s essential oil (CEO) shows antimicrobial activity [16,17] against *Escherichia coli* [18], *Staphylococcus aureus* [19] and *Bacillus subtilis* [20]. Many studies on CEO obtained from coriander seeds have recognized linalool as the most representative compound, and many of CEO’s important biological activities, such as anticancer, anti-inflammatory, and antioxidant, have been ascribed to this molecule [21]. Anticarcinogenic [22], antioxidant [23] and anti-diabetic activities [24] have been attributed to various coriander extracts.

**Figure 1:**
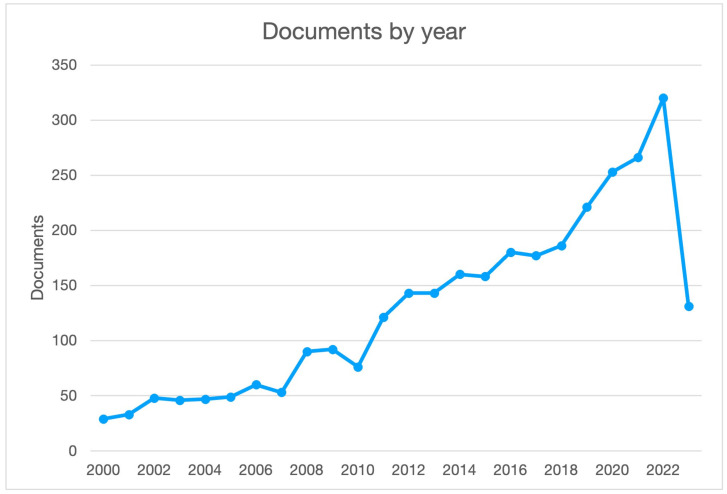
The number of papers published since 2000 (source Scopus) about “coriander”.

Starting from the beginning of this century, more than 3000 papers about “coriander” have been published (Scopus source) and more than 2000 only in these last 10 years. The rising number of published papers clearly shows the importance of this spice. Most of these papers deal with the preparation and the analysis of the essential oil (1002); conversely, only 343 are about polyphenols (Figure 1).

While the essential oil composition and its biological activities have been widely studied [20,25,26,27], the polyphenolic fraction of coriander still needs to be thoroughly described.

This review aims to update the polyphenolic composition of coriander seeds and leaves, outlining structures and biological properties against some inflammation-related diseases.

## 2. Coriander Phenolics

Recent studies have highlighted the presence of various secondary metabolites in *Coriandrum sativum*. The most important components found in coriander fruits are essential oil and fatty oil. Sterols, terpenoids, tocols are also present. Coriander vegetative parts, such as leaves and stems, present the greatest nutritional and health-promoting benefits due to their relatively high polyphenol content [28,29].

It is well known that many biological activities of plant extracts can be primarily attributed to the presence of phenolic compounds [30], which represent a large class of secondary plant metabolites with interesting biological properties. They are yellow, red, blue and purple pigments and are essential compounds in plant-animal and plant-microorganism relationships [31]. In plants, they can act as phytoalexin protective agents against UV light and play a crucial role in plant growth and reproduction [32,33]. In response to environmental and oxidative stress and free radical production, these compounds undergo biosynthesis and accumulation in the vegetative parts of plants [34]. Polyphenols, a major subgroup of phenolic compounds, are widely distributed throughout the plant kingdom [35,36] and they are known to possess a wide range of biological activities, such as the antioxidant that prevents the lipid peroxidation damage, the anti-inflammatory and the anticancer ones [32].

Different studies have shown that the amount and profile of phenolics in coriander extracts depend on several factors, such as their place of origin, solvent and the methodology used for their extraction.

Polyphenols from different parts of the plants show different profiles. For example, in 2011, Sriti et al. studied the polyphenolic content of coriander seeds coming from Tunisia and Canada and find that the Canadian sample is richer in polyphenols (15.16 mg gallic acid equivalents (GAE)/g vs. 12.10 mg GAE/g) [37]. Later (2012), the same authors study the polyphenolic content in coriander whole fruit, pericarp and seeds. They identify several phenolic acids, such as gallic, ferulic, caffeic, chlorogenic, syringic, vanillic, *p*-coumaric, rosmarinic and cinnamic acids, as well as some flavonoids, i.e., quercetin-3-rhamnoside, luteolin, coumarin, quercetin, rutin, apigenin and amentoflavone [38]. Chlorogenic acid, *p*-hydroxybenzoic acid, syringic acid and ferulic acid are absent in the pericarp extract. The only flavonoids present in all the extracts are rutin, luteolin and amentoflavone, while quercetin and apigenin are found only in the whole fruit.

Nambiar et al. (2010) [39] study the polyphenolic profile of coriander leaves and show the presence of vanillic, ferulic and *p*-coumaric acids. Regarding flavonoids, they find quercetin, kaempferol and acacetin.

Due to the importance of polyphenols for coriander biological activity, several studies have been proposed in the last few years to increase their production.

It has been shown that the preharvest treatment of coriander leaves with malic, oxalic and acetylsalicylic acids increases the polyphenolic content and hence the antioxidant activity of coriander extract [40].

Kianersi et al. (2022) [41] report that a notable plant hormone, methyl jasmonate (MeJA), has been shown to impact the accumulation of these secondary metabolites by modulating signal transduction and defence gene expression. The study by Kianersi et al. (2022) [42] further investigates the effects of MeJA on the expression of key genes involved in linalool and γ-terpinene biosynthesis, phenolic compound accumulation and linalool content in two Iranian *Coriandrum sativum* genotypes (Mashhad and Zanjan). The study results indicate that MeJA treatment enhances the expression of the linalool synthase (CsLINS) and γ-terpinene synthase (CsγTRPS) genes, leading to the increased biosynthesis of linalool and total phenolic content in the two genotypes.

The use of microbial biostimulants to improve the yield and quality of vegetable crops has been gaining increasing attention in recent years and EU Regulation 2019/1009 allows the use of some microbial biostimulants in Europe [43]. Jiménez-Gómez et al. (2020) [34] study the increase in total phenolic content of *Coriandrum sativum* L. after use as a biofertiliser of *Bacillus halotolerans*. The researchers find that by applying the biofertilizer, the total phenolic content in the coriander plants increases, resulting in a significant increase in the activity of antioxidant enzymes such as superoxide dismutase, catalase and peroxidase, potentially leading to improved antioxidant activity, which would be beneficial for human health.

In a related study, Faizan (2019) [44] aims to evaluate the impact of arbuscular mycorrhizal (AM) fungi and *Azotobacter* on alleviating cadmium (Cd)-induced growth reduction and the activity of antioxidants in *C. sativum* L. This study finds that the plant’s growth is significantly reduced because of Cd toxicity; however, AM fungi and *Azotobacter* are able to decrease the growth reduction. Furthermore, this study also finds that the application of AM fungi and *Azotobacter* increases the activity of antioxidants such as catalase (CAT) and superoxide dismutase (SOD) in the plant, suggesting that AM fungi and *Azotobacter* are beneficial for the growth and health of *C. sativum* L. as they can reduce the adverse effects of Cd toxicity and enhance the activity of antioxidants within the plant.

## 3. Extractive Methods: Traditional and Innovative

The study of nutraceuticals extracted from plants has recently gained increasing attention due to their potential health benefits, such as antioxidant and anti-inflammatory properties [45].

Conventionally, maceration, percolation and digestion techniques have been used; however, they have several limitations, such as high time and solvent consumption and the potential for heat-sensitive bioactive compounds to degrade [46,47]. All the extraction techniques are strongly influenced by several factors, such as extraction duration, solvent-to-plant ratio, and granulometry of the extracted sample [48].

As the research into natural products continues to evolve, advanced extraction methods have become crucial in the production of bioactive plant extracts [49]. It has been reported that the extraction process plays a critical role in determining the quality and yield of plant extracts [50], and it is essential to choose the suitable solvent system for maximum recovery of total phenolic content (TPC), total flavonoids (TF) and other antioxidant compounds [51,52].

The phenolic content and antioxidant properties of *C. sativum* are evaluated by Muñiz-Márquez et al. (2014) [30] using solid-liquid extraction with 35% aqueous ethanol by reflux-heat, resulting in a phenolic content of 1.38 mg GAE/g dry plant material (DW). Demir et al. (2020) [53] find a difference in the total polyphenols content expressed as mg GAE/g dry weight (DW) when coriander is extracted using 80% aqueous methanol or ethanol. The first solvent extracts 4.2 mg GAE/g DW, while the latter only 2.1 mg GAE/g DW.

In recent years, researchers have aimed to improve the extraction of biological active compounds from coriander by employing innovative methods, such as supercritical fluid extraction (SFE), subcritical water extraction (SWE), ultrasound-assisted extraction (UAE), microwave-assisted extraction (MAE), solid-phase extraction (SPE) and rapid solid–liquid dynamic extraction (RSLDE) [47].

In their study, Palmieri et al. (2020) [54] compare the maceration, Soxhlet, UAE and RSLDE extraction techniques for their effectiveness on the chemical composition and antioxidant activity of *C. sativum*, thyme and hemp extracts. They find several organic acids in the ethanol extract, like gallic, *p*-hydroxybenzoic, syringic, chlorogenic, ferulic and caffeic acids and flavonoids such as luteolin and apigenin. UAE does not extract gallic, *p*-hydroxybenzoic or syringic acids. On the other hand, common maceration extract shows the presence of only gallic, *p*-hydroxybenzoic, chlorogenic, syringic and ferulic acids and only apigenin among the flavonoids. The only extract that contains all phenolics is the one obtained using Soxhlet and refluxing for 6 h. Furthermore, results indicate that while the Soxhlet extraction method (6 h) results in the highest phenolic content for *C. sativum* (24.36 mg GAE/g DW), the RSLDE technique (2 h) shows higher extract activity in all spectrophotometric assays (except for DPPH where the highest antiradical activity is obtained through UAE extraction at 219.95 mg Trolox equivalents (TE)/g DW). The authors find that maceration is the least effective technique for extracting polyphenols and for antioxidant activity. They conclude that RSLDE and UAE are efficient methods for extracting phenolic compounds maximising the antioxidant activity and reducing solvent and energy consumption.

Zeković et al. extensively study the influence of the extraction method on the yield and composition of coriander. In 2014, they use subcritical water extraction (SWE) of antioxidant compounds from coriander seeds [55] and later optimise this procedure [56]. The optimal extraction conditions are found at a temperature of 100.5 °C, a pressure of 87.6 bar and an extraction time of 10 min, resulting in a maximum phenolic content of 1001 mg/100 g DW. The extract contains various phenolic compounds, including flavonoids, phenolic acids and other substances [56]. In 2015, they separate polar and non-polar compounds using a sequential supercritical carbon dioxide (SC-CO_2_) extraction and UAE with 70% ethanol. The results show that SC-CO_2_ is effective in extracting non-polar fractions, while UAE with 70% ethanol is effective in extracting polar fractions [57]. The combination of SFE and UAE is a promising approach for sequentially extracting non-polar and polar fractions from coriander seeds.

It is well known that the smaller particle size, which allows a more intimate contact between the plant material and the solvent, leads to higher extraction rates [58]. As such, Zeković (2015) [57] find that the reduction of particle size increases the amount of UAE-extracted polyphenols, but not using SFE, as it seems that this methodology can overcome the particle-size issue.

The disparities in total phenol (TP) results across different studies examining *C. sativum* can also be attributed to different varieties of plant material, cultivar, origin and time of harvesting.

Msaada et al. (2017) [7] evaluate the TP, TF and antioxidant activity in coriander extracts obtained by methanolic extraction, and the results show TP levels ranging from 94 to 109 mg GAE/100 g DW, depending on the variety of coriander used. However, these values are lower than those obtained using UAE and 70% ethanol and water (222.08 to 308.55 mg GAE/100 g DW), as reported by Zeković et al. in 2015 [57], and higher than those reported by Tirfan et al. in 2020 [59], using UAE and methanol at 60 °C for 30 min (3.80 mg GAE/100 g DW).

Gallo et al. (2010) [60] also compare MAE and UAE extraction of coriander seeds using 50% ethanol as the extraction solvent. They obtain an 82.01 and 41.81 mg GAE/100 g DW, respectively, which is a significantly lower TP content despite using similar extraction conditions to Zeković et al. (2015) [57] (1:10 sample-to-solvent ratio and extraction time of 30 min).

Mechchate et al. (2021) [61], starting from defatted seeds, prepare a 70% methanol extract using the UAE method, and find nine phenolics: vanillic, chlorogenic acids, catechin, epicatechin, epicatechin gallate, gallocatechin, oleuropein and rutin.

## 4. Antioxidant Activity of Coriander Extracts

The most characteristic activities of polyphenols are the antiradical and antioxidant ones. The good amount of polyphenols in *C. sativum* extracts makes it a suitable reducing agent, lipid peroxidation inhibitor, free radicals scavenger and quencher of singlet oxygen [62].

Besides the solvent and the extraction methodology, several other factors can influence the antioxidant activity of an extract. One of these is the chosen drying method of the plant material. Different drying conditions, such as time and temperature, can influence the stability of secondary metabolites and, hence, can affect the biological activity of extracts. It has been observed that increasing the drying temperature from 40 °C to 80 °C causes a loss of polyphenol content in coriander extracts, possibly due to the decomposition of unstable polyphenols [63]. On the other hand, the authors observe an increase in the polyphenol content drying at the temperature of 120 °C. This last result has been explained by the formation of Maillard reaction products [64] or by the release of bound phenols [65]. Mouhoubi (2022) [63], in his study, tests microwaves (MW) for drying coriander and other spices, observing that this procedure can reduce drying time compared to conventional convective drying. Furthermore, comparing drying at 40 °C and 100 W (power MW), he observes an increase in hydroxybenzoic acids (202.01 vs. 289.81 mg/100 g DW) but not in hydroxycinnamic acids and quercetin. The increase in polyphenols led to an increased antioxidant activity evaluated using FRAP and DPPH methods.

Wangensteen et al. (2004) [66], in the previously cited study, demonstrate a positive correlation between the extracted polyphenols and the antioxidant activity, which can play an important role in ameliorating several conditions, such as liver and kidney function after radiation-induced stress [67].

Laboratory studies involving Wistar rats have demonstrated the ability of *C. sativum* extract to mitigate oxidative stress. The administration of coriander extract, which was rich in phenolics and carotenoids in both etheric and aqueous forms, was found to result in a decrease in liver and plasma thiobarbituric reactive substances after 60 days of treatment [68]. The antioxidant properties of coriander are mainly attributed to its high levels of phenolics, including caffeic acid, protocatechuic acid and glycitin, with 2.734 mg of catechin equivalents per 100 g of dry samples [67]. The polyphenolic compounds in coriander even show a protective effect against H_2_O_2_-induced oxidative stress in human lymphocytes [69]. Furthermore, the hydroalcoholic seed extract of *C. sativum* has been studied for its ability to mitigate lead-induced oxidative stress in different regions of the rat brain, including the hippocampus (a crucial centre for learning and memory), the striatum (involved in motor control) and the cerebellum (responsible for coordination and balance) [70].

In another study, both aqueous and ethanolic coriander stem and leaves extracts are studied for their antioxidant activities, with fresh leaves demonstrating a superior reducing activity when compared to the other extracts [71]. These results are comparable to the standard drug “Silymarin” in increasing protective enzymes, including superoxide dismutase (SOD), catalase (CAT), glutathione peroxidase (GPx), glutathione reductase (GR) and glutathione-S-transferase [72,73] which demonstrates the antiperoxidative effect of polyphenols.

## 5. Anti-Inflammatory Activity of Coriander Polyphenols

Oxidative stress is closely related to inflammation and hence to several chronic diseases, such as neurodegenerative and cardiovascular diseases, cancer and arthritis. Phenolic compounds have exhibited anti-inflammatory activity [74]. Mechchate et al. (2021) [75] find that a UAE-methanol extract from coriander seeds can protect against carrageenan-induced inflammation. The same extract has an anti-diabetic activity. Coriander hydroalcoholic extract (10% water) from dried leaves is evaluated for its anti-inflammatory activity in carrageenan-induced pleurisy and croton oil-induced ear oedema tests. The extract shows anti-inflammatory activity in the pleurisy test, but no leukocyte migration is observed. The topical application of the extract decreases ear oedema in mice. These results confirm the anti-inflammatory activity of coriander extract [76].

Nitric oxide (NO) is a potent vasodilator but also plays an important role in inflammation pathogenesis. Coriander polyphenols can reduce inflammation by acting as NO radical scavengers, stabilising membranes and inhibiting protein denaturation [77], and reducing inflammatory and pro-inflammatory agents, such as PLA2, COX2, IL-6, IL-1α and TNF-α [78].

**Figure 2:**
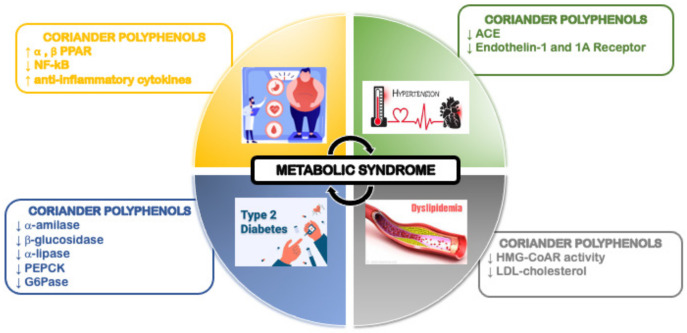
Importance of coriander polyphenols against metabolic syndrome.

The antioxidant and the anti-inflammatory activities of coriander polyphenols can help in the prevention of obesity, hypertension, dyslipidemia and type-2 diabetes and, hence, in the prevention of metabolic syndrome (Figure 2).

## 6. Coriander and Obesity

Obesity is a complex condition associated with numerous health issues, including cancer, diabetes mellitus, cardiovascular disease and chronic kidney disease [79]. The pathogenesis of obesity and metabolic syndrome is characterised by a chronic inflammatory response attributable to the infiltration of macrophages into adipose tissue, and the consequent release of the pro-inflammatory cytokines, COX, and iNOS [80]. As such, the quantification of visceral adipose tissue and hepatic mass represent key measures for assessing the extent and severity of metabolic dysregulation, which are integral to the development and progression of metabolic disorders [81].

It has been reported that polyphenol-rich spices, such as coriander, can activate PPAR-α and -β, inhibit the activation of NF-kB and increase the expression of anti-inflammatory cytokines [82].

Coriander polyphenols can help to ameliorate obesity and metabolic syndrome. Treating rats fed with a diet rich in cholesterol and triglycerides with coriander seeds leads to a hypolipidaemic effect. Furthermore, the authors observe an increase in the hepatic metabolism of cholesterol to bile acids that are faecally excreted [83]. However, research into broiler chicks has demonstrated that including whole coriander seeds in their diet can significantly improve growth performance, body weight, feed intake and conversion ratio [84].

Mexican researchers administered *C. sativum* seed powder or chia (*Salvia hispanica*) powder to volunteers for two months and both to a third group. The volunteers that were supplemented with coriander powder showed an increased antioxidant capacity and a decrease in glucose and cholesterol levels. The group treated with chia had decreased cholesterol and triglycerides while the third had decreased glucose, cholesterol and triglyceride levels. All of the subjects lost weight [85].

At the cellular level, adipogenesis, or the formation of fat cells, is a primary characteristic of obesity [86]. Although adipocytes are a necessary energy store, excessive triglyceride loading can cause inflammation, increase plasma triglyceride concentrations, and limit fat mobilisation, leading to potentially toxic ectopic fat deposition [87]. To address this issue, researchers have been exploring the use of phytochemicals such as quercetin, epigallocatechin-3-gallate, resveratrol, caffeic acid, and gallic acid to mobilise triglycerides with low toxicity [87,88]. Notably, extracts of *C. sativum* have been shown to decrease triglyceride formation in 3T3-L1 cells and ameliorate insulin resistance and adipocyte hypertrophy [89]. Furthermore, *C. sativum* extracts have demonstrated the ability to reduce lipid accumulation and prevent adipogenesis at higher dosages [90] due to their significant amounts of flavonoid and phenolic components with antioxidant potential [91].

## 7. Coriander and Diabetes

Chronic and systemic inflammation can cause insulin resistance (IR) and other problems leading to type 2 diabetes (T2D) [92]. It has been reported that polyphenols can have a beneficial effect in fighting T2D, enhancing glucose uptake in muscles and adipocytes [93].

The anti-diabetic properties of coriander polyphenols have been largely tested. Mechchate et al. (2021) [75] find that a polyphenol-rich coriander (PCS) extract plays an important role in controlling hyperglycemia in diabetic mice. The authors demonstrate that this anti-diabetic property is related to the antioxidant and anti-inflammatory activities of the extract. In another study, researchers compare the effects of *C. sativum* with the standard anti-diabetic drug, Metformin, in diabetic rats induced by Streptozotocin. Blood sugar levels (BSLs) significantly increase after Streptozotocin administration but decrease upon the administration of *C. sativum* seed extract, further reducing HbA1C levels. However, the reduction is more prominent with chronic administration, although Metformin shows a more significant reduction in BSL than the test drug. The study concludes that *C. sativum* seed extract at a dose of 40 mg/kg BW (Body Weight) shows anti-hyperglycaemic activity in Streptozotocin-induced diabetic rats, which suggests its potential as an anti-diabetic agent and a possible dietary supplement [94].

*Trigonella foenum-graecum* L. (fenugreek) is a well-known anti-diabetic plant [95,96]. Yella et al. (2019) [97] compare the anti-diabetic activity of fenugreek and coriander extracts in Alloxan-induced diabetes in Wistar albino rats. Evaluating different parameters, such as hepatic and renal biomarkers, they find that the administration of the extracts for 21 days decreases blood glucose, cholesterol, triglycerides, and “bad” lipoproteins levels. The ethanol fenugreek extract has a significant anti-hyperglycaemic activity at 100 mg/kg BW, while the coriander seed ethanolic extract is active at 200 mg/kg BW. It is interesting to find that the polyherbal formulation (1:1 ratio of the two extracts) shows anti-diabetic activity comparable to Glibenclamide [97].

## 8. Conclusions

Phenolic compounds found in plants have potent antioxidant and anti-inflammatory properties and are potentially useful to improve human health. A literature analysis of the polyphenols produced by *Coriandrum sativum* L. shows that they could protect cells and the body from oxidative stress, which can contribute to ageing and degenerative diseases. In view of these activities, coriander is a potential complement for the treatment of metabolic syndrome and related problems. However, it has been demonstrated that the profile and amount of the extracted polyphenols depend on several factors such as plant species, growing conditions and harvesting time; also, the methodology and solvent used for extraction can play an important role. We can conclude that coriander seeds and leaves have the potential to ameliorate obesity and its associated risk factors due to their powerful antioxidant and anti-inflammatory activities.

## Data Availability

Not applicable.
